# Global proteomic analysis of insulin receptor interactors in glomerular podocytes

**DOI:** 10.12688/wellcomeopenres.16072.1

**Published:** 2020-08-26

**Authors:** Salman B. Hosawi, Jonathan D. Humphries, Richard J. Coward, David Knight, Martin J. Humphries, Rachel Lennon

**Affiliations:** 1 Wellcome Centre for Cell-Matrix Research, University of Manchester, Manchester, M13 9PT, UK; 2 Department of Biochemistry, King AbdulAziz University, Jeddah, Saudi Arabia; 3 Academic Renal Unit, University of Bristol, Bristol, UK; 4 Biomolecular Analysis Core Facility, University of Manchester, Manchester, M13 9PT, UK; 5 Department of Paediatric Nephrology, Royal Manchester Children’s Hospital, Manchester, UK

**Keywords:** Podocytes, insulin signalling, insulin receptor, insulin resistance, DCDC2, mass spectrometry-based proteomics

## Abstract

**Background:** Insulin signalling contributes to diverse cellular activities including protein synthesis, proliferation and cell survival. Insulin resistance describes the inability of cells to activate the insulin signalling pathway effectively; leading to pathological effects in multiple organ systems including the kidney. In diabetic kidney disease, there is progressive glomerular dysfunction and recent studies have demonstrated that the kidney podocyte is a direct target for insulin action. In this study we defined the literature-based insulin receptor (INSR) interactome and utilised an unbiased proteomic approach to examine INSR interactors in podocytes.

**Methods:** Human podocytes expressing the INSR were characterised under basal and insulin resistant conditions. The INSR was isolated by whole cell immunoprecipitation following a time course stimulation of 2, 7, and 15 minutes with of 100nM insulin. The resulting INSR complexes were analysed by label-free mass spectrometry (MS) to detect protein interactors.

**Results: **We identified 27 known, direct INSR interactors in addition to novel interactors including doublecortin domain-containing protein 2 (DCDC2). The interaction of DCDC2 with the INSR was confirmed by immunoprecipitation and immunofluorescence, and under insulin resistant conditions, DCDC2 had increased association with the INSR. siRNA knockdown of DCDC2 in podocytes resulted in cell morphological change and altered INSR localisation.

**Conclusion:** This study provides insight into the complexity of INSR interactors in podocytes and highlights DCDC2 as a novel INSR binding protein. Involvement of this novel interactor in insulin signalling and podocyte biology may explain how insulin resistance alters morphology and integrity of the glomerular filtration barrier.

## Introduction

Insulin plays a key role in energy homeostasis and insulin signalling contributes to diverse cellular activities including protein synthesis, proliferation and cell survival ^1^. Abnormal physiological conditions and disease processes can interfere with the insulin signalling pathway leading to complete or partial loss of downstream effects. This state is known as insulin resistance ^2^, which describes the inability of cells to activate the insulin signalling pathway effectively leading to pathological effects in multiple organ systems including muscle, liver, and kidneys ^3, 4^. Insulin resistance is associated with high levels of the free fatty acid palmitate and palmitate has been shown to induce cellular insulin resistance in a number of cell types ^5– 7^.

Type 2 diabetes has a huge global prevalence with more than 500 million individuals affected worldwide ^8^. Insulin resistance is a hallmark feature and poor glycaemic control can lead to multiple secondary complications including diabetic kidney disease (DKD). 30–40% of individuals with either type 1 or type 2 diabetes develop DKD, which is a leading cause of chronic kidney disease worldwide ^9^. In the early stages of DKD the kidney glomerulus undergoes hypertrophy and there is progressive deposition of extracellular matrix, associated with thickened basement membranes and mesangial matrix expansion ^10^. With progression there is podocyte foot process effacement leading to albuminuria, which is an early clinical feature of DKD. A number of studies have demonstrated that among the different cell types in the kidney, the podocyte is a direct target for insulin action, and the abrogation of insulin signalling specifically in these cells can cause progressive kidney damage and CKD ^5, 11, 12^.

Podocytes are terminally differentiated, specialised epithelial cells and are located on the outer surface of glomerular capillaries. These cells are known for their unique morphology featuring large cell bodies, primary processes, and interdigitating foot processes. Podocytes are insulin-sensitive cells and capable of rapidly transporting glucose via the transporter GLUT4 ^3^. Furthermore, deletion of the insulin receptor (INSR) in podocytes demonstrated the key role of this signalling axis as mice developed albuminuria with associated podocyte foot process effacement and altered extracellular matrix, mimicking features of DKD in humans ^11^. The unique morphology of podocytes is influenced by tight regulation of cytoskeletal components ^13^ and therapeutic targeting of actin-dependent dynamin oligomerisation has been proposed as a therapeutic strategy ^14^. Whilst a number of studies have focussed on defining podocyte protein interaction networks ^15, 16^ to elucidate mechanisms of disease, a primary focus on the INSR is lacking.

This study utilised mass spectrometry (MS)-based proteomics to examine INSR interactors in podocytes under healthy conditions. The primary aim was to identify known and novel INSR interactors and the secondary aims were to: 1) examine novel interactors in the context of palmitate-induced insulin resistance and 2) relate INSR interactor pathways to podocyte morphology and function.

## Methods

### Antibodies

Antibodies used were against mouse anti-insulin receptor (INSR beta Monoclonal Antibody (CT-1), Thermo Fisher Scientific, catalogue number: MA5-13778), ^17^, rabbit anti-AKT (Polyclonal antibody, Cell Signalling Technology, catalogue number: 9272) ^18^, rabbit anti- AKT [pS473] (Polyclonal antibody, Cell Signalling Technology, catalogue number: 9271) ^19^, rabbit anti- p44/42 MAPK (Erk1/2) (Polyclonal antibody, Cell Signalling Technology, catalogue number: 9102) ^20^, rabbit anti- Phospho-p44/42 MAPK (Erk1/2) (Thr202/Tyr204) (Polyclonal antibody, Cell Signalling Technology, catalogue number: 4370) ^19^, mouse anti-IRS1 (IRS-1 monoclonal antibody, Cell Signalling Technology, catalogue number: L3D12) ^21^, rabbit anti-phospho-IRS1 (Tyr608) mouse/ (Tyr612) human Antibody (Polyclonal antibody, Merck Millipore, cat# 09-432) ^22^, goat anti-DCDC2 (polyclonal antibody, Abcam, catalogue number: ab45868, western blotting dilution [1:1000], Immunofluorescent imaging [1:250]), mouse anti-Actin (monoclonal antibody, Sigma-Aldrich, catalogue number: A4700, Clone AC-40) ^23^, mouse anti-Beta1 integrin (monoclonal antibody, Sigma-Aldrich, catalogue number: MAB1965, a.a. 82-87, clone JB1A) ^24^. Alexa Fluor 488 Phalloidin molecular probe was used for detecting actin filaments (Thermo Fisher Scientific, catalogue number: A12379) ^25^ and tubulin was detected with rat anti-alpha tubulin (YL1/2) (monoclonal antibody, Thermo Fisher Scientific, catalogue number: MA1-80017) ^26^ or mouse anti-beta tubulin (monoclonal antibody, Thermo Fisher Scientific, catalogue number: MA5-16308). Secondary antibodies conjugated to AlexaFluor 680 (Donkey anti-rabbit polyclonal antibody, Life Technologies, catalogue number: A10043) ^27^ were used for immunofluorescence and IRDye 800 (Goat polyclonal antibody anti-mouse, Rockland antibodies and assays, catalogue number: 610-145-121) ^28^ were used for western blotting.

### Cell culture

Conditionally immortalised mouse and human podocytes were kindly provided by Moin A. Saleem and Richard J. Coward and were cultured as previously described ^29^. Briefly, podocytes between passage 10 and 16 were grown in uncoated tissue culture plates in RPMI-1640 medium with glutamine (R-8758; Sigma-Aldrich) supplemented with 10% (v/v) foetal calf serum (Life Technologies) and 5% (v/v) ITS (I-1184; Sigma-Aldrich; 1 ml/100 ml). Initially cells were cultured at 33°C for proliferation and then switched to 37°C for 10–14 days for differentiation into mature podocytes. Insulin stimulation was based on previously described work ^30^. Briefly, cells were cultured in RPMI-1640 medium without ITS or growth factors for 16 hours, followed by 100 nm insulin stimulation for 15 minutes as a default setup, or for different time intervals for time-course experiments. The insulin-containing media was then removed, and the cells washed by ice-cold PBS. Inducing insulin resistance in podocytes followed a previously described method with modifications ^5^. Briefly, the standard culture medium was supplemented with 5% (w/v) fatty acid free bovine serum albumin (BSA), 750 µM palmitate, and 1%(v/v) ethanol. The control cells were cultured in the same medium without palmitate but with 1%(v/v) ethanol. Cells were incubated with palmitate for 24 hours and prior to insulin stimulation cells were serum starved in RPMI-1640 medium without ITS or growth factors and supplemented with 5% (w/v) fatty acid free BSA, 750 µM palmitate, and 1% (v/v) ethanol or without palmitate for control cells.

### siRNA knockdown

ON-TARGETplus® DCDC2 siRNA SMARTpool and non-targeting siRNA were obtained from (Cat#: L-020868-02-0050, Dharmacon, Colorado, USA). Podocytes were grown to approximately 90–95% confluence and RNAi-mediated knockdown was performed using Lipofectamine 2000 (Invitrogen) and specific siRNA oligonucleotides in the presence of Opti-MEM according to manufacturer’s instructions. The siRNA oligonucleotides and Lipofectamine 2000 were diluted independently in Opti-MEM at a ratio of 1:5 per well for a 6 well tissue culture plate, and then incubated at room temperature for 5 minutes. The two components were then combined and further incubated at room temperature for 40 minutes. The media was removed from cells and the transfection mixture added in a 1:1 ratio with complete cell culture media. The cells were incubated for 4–6 hours before converting to normal growth medium.

### 2-NBDG glucose uptake

These experiments using a fluorescent indicator for direct glucose uptake were based on the previously described method with modifications ^31^. Podocytes were cultured and differentiated in 100mm plates, and a single plate was used per condition. Cells were serum starved for a minimum of 2-12 hours and then supplemented with or without 200 µM 2-[N-(7-nitrobenz-2-oxa-1,3-diazol-4-yl) amino]-2-deoxy-D-glucose (2-NBDG) dissolved in serum free medium for 1 hour at 37°C. Cells were then washed twice with ice-cold PBS, and lysed in 100 μl ice-cold lysis buffer. 70 μl of the cell lysate was used for assessing the 2-NBDG uptake in cells using a fluorescent plate reader, while the remaining 30 μl was used for quantifying the total protein of the samples using bicinchoninic acid assay (BCA assay) (Thermo Fisher catalogue number: 23225).

### Immunoprecipitation of the INSR and detection by mass spectrometry

Protein G magnetic dynabeads® (Thermo Fisher catalogue number: 10004D) were aliquoted to Eppendorf tubes, washed once with PBS, and re-suspended in 0.02% (v/v) PBS-T (PBS, 0.02% Tween) buffer. The mouse anti-insulin receptor antibody (beta subunit) (clone CT-1; Thermo Scientific) was added to the beads and incubated for 16 hours in 4°C with rotation. Beads were washed twice with 0.02% (v/v) PBS-T buffer, and then re-suspended in 630 μl [0.15 M NaCl (pH 7.8)]. 2 mg of bis(sulfosuccinimidyl)suberate (BS3) crosslinker was re-suspended in 70 μl MilliQ H _2_O and then added to the beads, and the beads were incubated for 2 hours on ice with mixing at an interval of 30 minutes. The BS3 crosslinker was quenched by adding 100 mM Tris buffer for 10 minutes at room temperature with rotation. The beads were washed twice with 0.02% (v/v) PBS-T buffer and stored at 4°C until needed. The cells were lysed in an immunoprecipitation buffer (ThermoFisher, Pierce™ IP Lysis Buffer catalogue number: 87787) supplemented with phosphatases and proteases inhibitors (ThermoFisher, Halt™ Protease and phosphatase Inhibitor Cocktail, EDTA-free catalogue number: 78445). The cell lysate was kept on ice for 30 minutes with vortexing every 10 minutes, and then centrifuged at 14000 rpm for 10 minutes at 4°C using a benchtop centrifuge. The supernatant was transferred to a clean tube and kept on ice, and the pellet was discarded. The pre-coated beads were added to the cell lysate and incubated for 2–4 hours at 4°C with rotation, and then the lysate was transferred to a new tube (the flow through, which is the unbound receptor fraction) and the beads were eluted in SDS sample buffer for 10 minutes at 95°C. The eluted complexes were transferred to a new tube, and the beads were discarded.

### MS sample preparation

As previously ^32^, for in-gel proteolytic digestion, gel lanes were cut into slices and each slice cut into ~1 mm ^3^ pieces. Gel pieces were de-stained three times with (50% (v/v) acetonitrile and 50% (v/v) 25mM NH4 HCO3) solution for 30 minutes to remove protein stain, and then dehydrated by immersing in acetonitrile followed by vacuum centrifugation for 1 hour. The proteins were reduced in 10 mM DTT, alkylated in 55 mM ioodoacetamide and then washed with alternating washes of 25mM NH _4_HCO _3 _and acetonitrile. The gel pieces were dehydrated using a vacuum centrifuge and then rehydrated and digested with sequencing grade trypsin (12.5 ng/μl in 25mM NH4 HCO3) at 37°C overnight. Peptides from the gel slices were collected in single wash of (99.8% (v/v) acetonitrile, and 0.2% (v/v) formic acid) and single wash of (50% (v/v) acetonitrile and 0.1% (v/v) formic acid). Peptides were desiccated in a vacuum centrifuge and re-suspended in 50 μl of (5% (v/v) acetonitrile and 0.1% (v/v) formic acid). To desalt peptides, each sample was resuspended in 5% (v/v) acetonitrile in 0.1% (v/v) formic acid followed by incubation with OLIGOTM R3 beads (Applied Biosystems, Paisley, UK). Bead-bound peptides were washed twice in 0.1% (v/v) formic acid, eluted by two washes in 50% (v/v) acetonitrile in 0.1% (v/v) formic acid, dried and resuspended in 5% (v/v) acetonitrile in 0.1% (v/v) formic acid.

### MS data acquisition, data analysis and data deposition

The analysis of the samples by the MS instruments was performed through the Biological Mass Spectrometry core facility at the University of Manchester. Peptides were analysed by liquid chromatography-tandem mass spectrometry (LC-MS/MS) using an UltiMate 3000 Rapid Separation LC (RSLC, Dionex Corporation, Sunnyvale, CA, USA) coupled to an Orbitrap Elite mass spectrometer (Thermo Fisher Scientific). Peptide samples were separated on an analytical column (250mm × 75 µm i.d., 1.7 µm particle size, bridged ethyl hybrid C18; Waters) over a 2 h program with a gradient of 92% A (0.1% formic acid in water) and 8% B (0.1% formic acid in acetonitrile) to 33% B, in 104 min at 300 nL min ^-1^. LC-MS/MS analyses were performed in data-dependent mode to allow automatic selection of peptides for fragmentation. Tandem mass spectra were extracted using extract_msn (Thermo Fisher Scientific) executed in Mascot Daemon (version 2.4; Matrix Science). Mascot Deamon is a software that automate the file search on the Mascot server, and this task can also be performed on the Matrix Science website with one file at a time. Peak list files were searched against a modified version of the Uniprot mouse database (version 3.70; release date, 3 May 2011), containing ten additional contaminant and reagent sequences of non-mouse origin, using Mascot server (version 2.2.06; Matrix Science) that can be accessed using Matrix science website. Carbamidomethylation of cysteine was set as a fixed modification; oxidation of methionine and hydroxylation of proline and lysine were allowed as variable modifications. Only tryptic peptides were considered, with up to one missed cleavage permitted. Monoisotopic precursor mass values were used, and only doubly and triply charged precursor ions were considered. Mass tolerances for precursor and fragment ions were 20ppm and 0.5 Da, respectively. MS datasets were validated using rigorous statistical algorithms at both the peptide and protein level implemented in Scaffold (version 3.6.5; Proteome Software). Protein identifications were accepted upon assignment of at least two unique validated peptides with ≥90% probability, resulting in ≥99% probability at the protein level. These acceptance criteria resulted in an estimated protein false discovery rate of 0.1% for all datasets. Alternatively, peptide quantification and protein identification over the time course of insulin-induced INSR interactions was based on the precursor ion (MS1) intensities as a measure of protein abundance using Progenesis QI™ software (version 4.0). The quantification was based on 3 peptides per protein, and the identification was based on 2 peptides per protein. Raw data with identification and quantification outputs from the MS analyses have been deposited at Figshare ^33^. The proteomic analysis tasks performed by Scaffold™ and Progenesis QI™ can be performed using MaxQuant, which is an open source software.

### Protein interaction network analysis

The insulin receptor interactome was built from the protein-protein interaction public databases STRING (version 10.5) and BioGRID (version 3.4). Briefly, the INSR interaction network from both databases was downloaded at default settings for each database in Tab-delimited format. Data merging was performed using Microsoft Access 2011, and the evidence used for evaluating the confidence of interaction scores were primely found in the BioGRID imported file. This evidence was reviewed to reassess and re-score the interactions in the new network. A numerical scoring system was derived based on the type of experiment and amount of evidence. A low confidence interaction was given a score between 0.1–0.3 based on 1 piece of evidence from: 1) affinity capture-mass spectrometry, 2) yeast two-hybrid screen. A medium confidence interaction was given a score between 0.4–0.6 based on 2-3 pieces of evidence from the same categories as low confidence but with the addition of: 1) affinity capture-immunoblot, 2) bioluminescence/fluorescence resonance energy transfer, 3) kinase activity, 4) phosphatase activity. A high confidence interaction was given a score between 0.7–0.9 based on more than 3 pieces of evidence from the same categories as low and medium confidence in addition to: 1) crystal structure reconstituted complex.

### Ingenuity pathway analysis

Data were analysed through the use of Ingenuity Pathway Analysis (IPA; QIAGEN Inc.) to identify pathways predicted to be active in control and insulin treated samples over the 2, 7 and 15 minute time course as previously described ^34^. Perseus and Reactome software can be used to perform similar analysis to IPA, and the terminologies and the enriched pathways are similar between these software with some differences in the level of the classification terms.

### Electrical cell-substrate impedance sensing assay

Electrical cell-substrate impedance sensing (ECIS) plates (Applied biophysics, 8W10E PET, catalogue number: 72010) were coated with extracellular matrix ligands type IV collagen (purified from human placenta, Sigma-Aldrich, catalogue number: C5533) and laminin (purified from human placenta, Sigma-Aldrich, catalogue number: L6274) (dissolved in 0.15 M NaCl) for 1 hour at room temperature, and then briefly washed with MilliQ H _2_O. The chambers were inserted into the ECIS instrument with serum-free media for 2 hours, and then the cells were seeded at equal numbers into the chambers and allowed to adhere for a minimum of 2 hours. Insulin or vehicle control were added directly to the wells without pausing the experiment, while supplementing the cells with full growth media required the removal of the plates from the instrument. The measurements were collected at 4000Hz according to the manufacturer instructions.

### Immunofluorescence and image analysis

Cells on coverslips were washed with PBS and then fixed with 4% (w/v) paraformaldehyde. Cells were permeabilised with 0.5% (v/v) Triton X-100 and blocked with 3% (w/v) BSA in PBS before incubation with primary antibodies. Coverslips were mounted and images were collected using a CoolSnap HQ camera (Photometrics, Tucson, AZ, USA) and separate DAPI/FITC/Cy3 filters (U-MWU2, 41001, 41007a, respectively; Chroma, Olching, Germany) to minimise bleed-through between the different channels. The images were collected using a Coolsnap HQ (Photometrics) camera with a Z optical spacing of 0.2μm. Images collected were viewed and analysed with Fiji (version 1.5) ^35^.

### Western blotting

All SDS-PAGE experiments were performed using NuPAGE® precast gels system (NuPage, Invitrogen) and the specifically designed apparatus by the same manufacturer. Protein samples were run into the gels at 150–200 volts for 40–60 minutes in 1x Bolt™ MES running buffer. Following SDS-PAGE, separated proteins were transferred onto nitrocellulose membrane (Whatman, Maidstone, UK). Membranes were blocked using casein blocking buffer (Sigma-Aldrich) for 1 hour at room temperature and probed with primary antibodies diluted in blocking buffer containing 0.05% (v/v) Tween-20 overnight at 4°C. The membranes were washed with PBS-Tween and incubated with species-specific fluorescent dye–conjugated secondary antibodies diluted in blocking buffer containing 0.05% (v/v) Tween-20. Finally, the membranes were washed in dim lightning, and then scanned using the Odyssey infrared imaging system (LI-COR Biosciences, Cambridge, UK) to visualise bound antibodies.

### Statistical analysis

All error bars represent standard error of the mean (SEM) unless stated otherwise. For statistical analyses, one-way analysis of variance (one-way ANOVA) was applied to compare means of more than two samples within the same experiment. The Student’s t-test was used, where appropriate, to directly compare between two samples. The clustering of the data used the Pearson uncentred assumption with complete linkage. The heat maps used the z-score (standardisation) to set the values within a manageable numerical range. The quantification of the proteomic data (ion intensity) was based on a minimum of three peptides, and the quantification was performed using Progenesis software (Progenesis QI, Nonlinear Dynamics). The statistical tests were performed using Microsoft Excel 2016 and GraphPad Prism (version 7.0a) The proteomic statistical analysis performed by Progenesis QI™ could be performed using MaxQuant and Perseus software.

## Results

### *In silico* analysis of known INSR interactors

**Figure 1.**
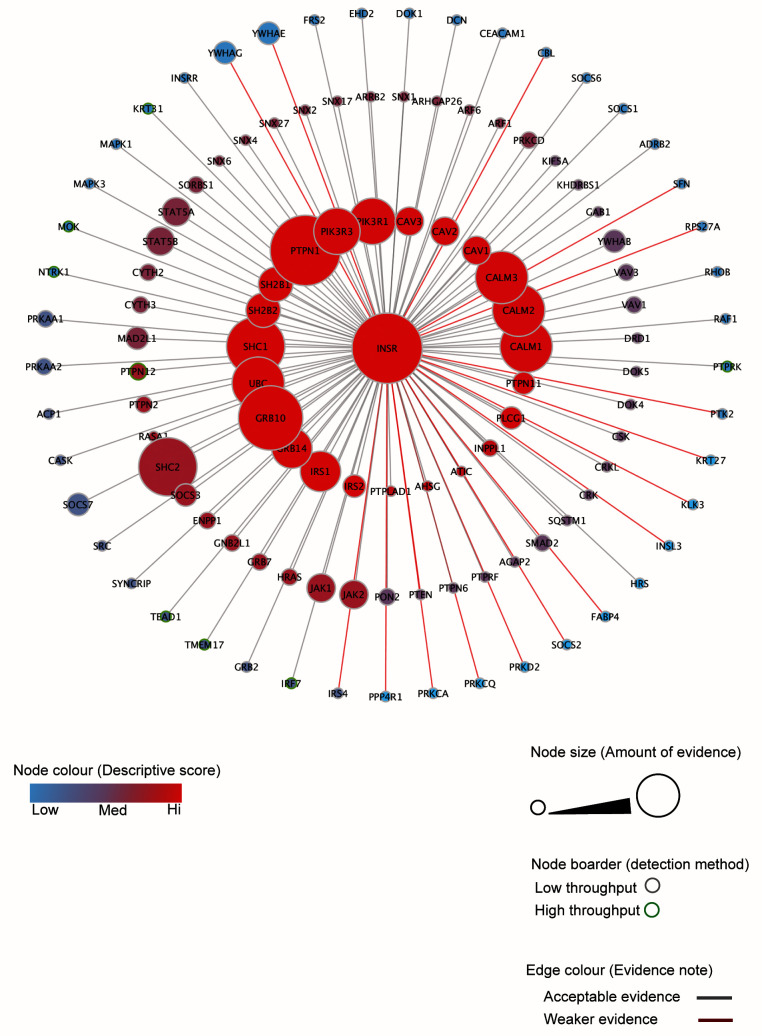
The *in silico* analysis of known INSR interactors. The analysis of the INSR interactome *in silico* based on protein-protein interactions obtained from two public databases (STRING (version 10.5) and BioGRID (version 3.4). INSR interactors were arranged in 3 consecutive circles according the level of confidence in supporting evidence of the interaction. A numerical scoring system was used to grade evidence on the basis of the type of experiment and the amount of evidence (see methodology). The node size reflects the amount of evidence and the node colour indicates the level of confidence score for the interaction. The node border indicates whether the interaction was detected in a low or high throughput experiment and the colour of the edges represents the level of confidence in the interaction.

To generate a comprehensive INSR interaction network, an *in silic*o INSR interactome was created by merging the INSR interactions from BioGRID and STRING databases and combining with a detailed review of the evidence associated with the BioGRID data ( Figure 1). The interaction search was across mammalian cell and tissue systems and included experimental evidence of interaction but excluded predicted interactions. The network was designed in a spoke style where each node represents an INSR binder, and the three consecutive spoke rings are arranged according to the strength of evidence in the databases and corresponding support from published literature. Cumulative evidence for the different INSR interactors in the network was manually re-evaluated and scored as described in the methods section. Furthermore, the generation of this network minimised the chances of missing some known INSR interactors by encompassing unique interactions that are found in one database but not the other. Gene Ontology (GO) analysis of the components of this network highlighted expected terms such as protein tyrosine kinase activity, receptor tyrosine kinase binding, and protein tyrosine phosphatase activity (Extended data Figure 1 ^33^). This network and its GO terms were used as a reference in the assessment of our subsequent proteomic analysis of INSR interactors in podocytes.

### Cultured podocytes are insulin sensitive

**Figure 2.**
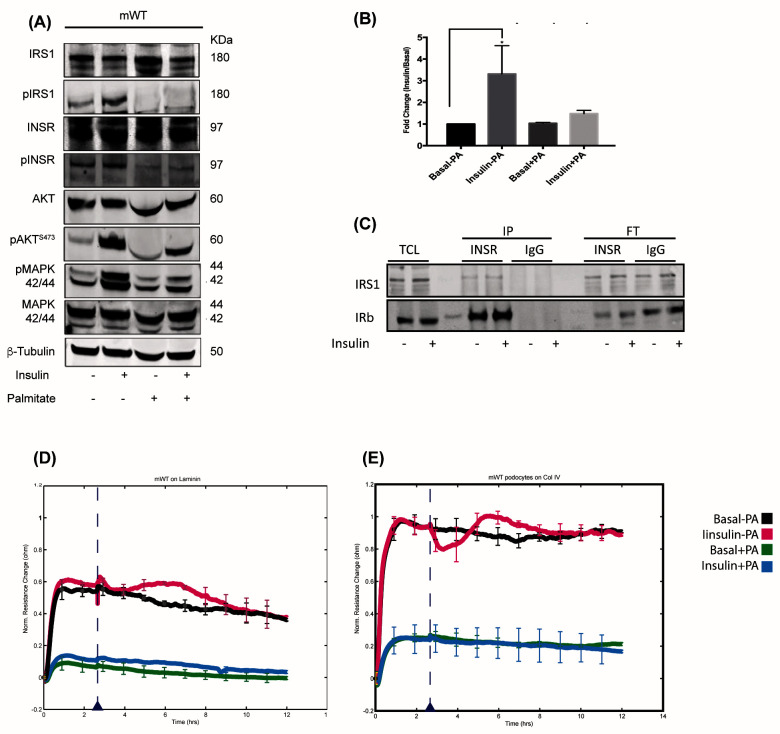
Podocytes are insulin sensitive. ( **A**) Cultured differentiated mouse wild-type (mWT) podocytes are insulin responsive and can activate the PI3K and MAPK pathway. In the presence of palmitate, the cells became insulin resistant with reduced activity of both pathways. ( **B**) Podocytes responded to insulin stimulation by initiating 2-NBDG uptake (a glucose analogue) up to three-fold compared to the basal condition, which was lost in the palmitate treatment. ( **C**) The insulin receptor (INSR) was isolated using immunoprecipitation, and the insulin receptor substrate 1 (IRS1) was co-immunoprecipitated with the receptor. ( **D** and **E**) The electrical resistance formed by the adhesion of podocytes to laminin and collagen and their formation of a monolayer-like layer measured by electrical cell-substrate impedance sensing (ECIS).

Wild type mouse podocytes were used to examine insulin induced INSR interactions by MS-based proteomics. To verify insulin sensitivity in these cells, we performed characterisation experiments. Western blotting demonstrated a good response to insulin stimulation (100 nM, 15 minutes), with increased phosphorylation of the INSR, and both the phosphoinositide-3 kinase pathway (PI3K) pathway (phospho-IRS1 and -AKT ^473^) and the mitogen-activated protein kinase (MAPK) pathway (phospho-MAPK ^42/44^) compared to the basal state ( Figure 2A). Furthermore, palmitate-induced insulin resistance resulted in the suppression of this response, in line with previously reported observations ^5^. Insulin stimulation also prompted glucose uptake in podocytes, as demonstrated by uptake of the glucose analogue 2-NBDG, and this response was reduced upon palmitate induced insulin resistance ( Figure 2B). Since the formation of the glomerular filtration barrier relies on interdigitating podocytes interacting with glomerular basement membrane (GBM) ligands, we used electrical cell-substrate impedance sensing (ECIS) to examine the barrier function of podocytes plated on either type IV collagen or laminin. We observed a response to insulin on both GBM ligands, where a drop in electrical resistance was recorded minutes after insulin stimulation followed by a recovery and stabilisation of the monolayer ( Figure 2D, 2E). Palmitate induced insulin resistance resulted in a much lower electrical resistance compared to basal conditions, and a complete loss of response to insulin stimulation. Together these findings confirm insulin sensitivity in cultured podocytes, with changes in cellular signalling, glucose uptake and barrier function. Furthermore, these responses were lost with palmitate induced insulin-resistance.

### Immunoprecipitation of the INSR and detection by mass spectrometry

The endogenous INSR was isolated from mouse podocytes using antibody-based immunoprecipitation (IP), where the receptor was targeted by antibodies recognising the intracellular (β subunit), ( Figure 2C). Insulin receptor substrate 1 (IRS1) was primarily used to monitor the co-IP of known INSR interactors by western blotting. Although the INSR and some interactors were detectable by western blotting, analysis by mass spectrometry yielded only low levels of the endogenous INSR. To scale up the isolation of the receptor, immortalised human podocytes overexpressing the human insulin receptor (hWT-IR) were used to isolate the INSR. As previously reported ^36^ our functional evaluation of the hWT-IR cells demonstrated insulin responsiveness as shown by the activity of PI3K and MAPK, and the 2-NBDG uptake; however, these cells did not become insulin resistant in the presence of palmitate (Extended data Figure 2A-B ^33^). Importantly, co-IP of the INSR and some of its known interactors was reproducible in the hWT-IR cells (Extended data Figure 2C ^33^), and the MS analysis of the INSR complex demonstrated the dominance of the INSR peptides. The MS analysis of the INSR complexes 15 minutes after insulin stimulation identified a number of known INSR interactors and to further improve the likelihood of detecting known INSR interactors in the proteomic analysis, the INSR was isolated after a time course of 2, 7, and 15 minutes of insulin stimulation (Extended data Figure 3 and Extended data Table 1 ^33^). This resulted in the detection of the INSR and 26 of its known interactors that were found in the INSR *in silico* network (Extended data Table 1 ^33^), and these 26 interactors were found across two independent MS experiments. The identification of known INSR interactors such as IRS1, IGF1, GRB10, CAV1 and PTPN1 increased our confidence in identified interactors in the proteomic dataset.

### Proteomic analysis of INSR complex revealed novel interactors

Protein identification and relative quantification over the time course of insulin-induced INSR interactions was based on the precursor ion (MS1) intensities as a measure of protein abundance, and the analysis was performed using Progenesis QI™ software. Quantification was based on 3 peptides per protein, and identification was based on 2 peptides per protein. The resulting protein list was analysed using four approaches: a) network analysis of the known INSR interactors to identify connections between these proteins and other proteins in the dataset, b) clustering analysis to identify patterns of recruitment, c) generation of INSR interactor groups based on identification confidence and increase following insulin stimulation and d) Ingenuity Pathway Analysis (IPA) analysis to identify significant terms and molecular functions associated with INSR interactors.

**Figure 3.**
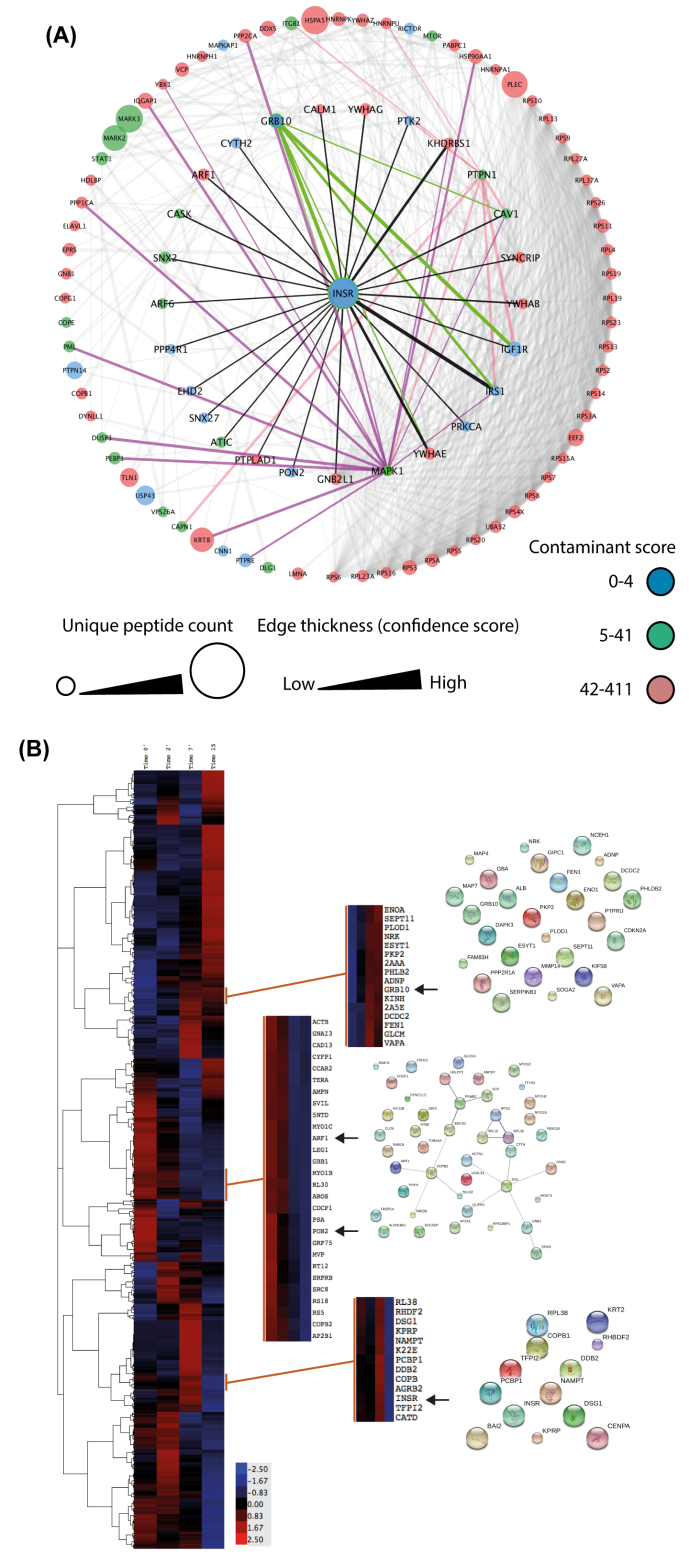
Proteomic analysis of INSR interactors in podocytes. ( **A**) A one-hop analysis of the INSR proteomic data linking the detected known INSR binders to other identified proteins in the dataset. The network was further mapped onto the Contaminant Repository for Affinity Purification to discriminate between background hits and potential novel candidates. ( **B**) A heat map of the proteins with a minimum of 2 unique peptides (Pearson uncentred and complete linkage). Clusters containing known INSR binders (arrows) were extracted with a minimum of (0.7) correlation score to discover complexes and pathways.

Firstly, the 26 known INSR interactors identified from the proteomic dataset and mapped onto the *in silico* INSR were inter-linked with the rest of the proteins within the dataset to discover new interactions. This approach was limited to 500 proteins per search using the STRING database. To highlight potential false positive identifications the resulting network was then searched against the Contaminant Repository for Affinity Purification ^5^ since immunoprecipitation can result in the co-isolation of non-specific interactions. The list was then sub-divided into three categories to express the chances of detecting background proteins as a percentage according to the total number of experiments in the list (total of 411). As such a protein with a score between 0-4 experiments would have a chance up to 1% of being a random protein, 5-41 would be up to 10% chance, and 42-411 would be up to 100% chance of a particular protein being a false positive being detected in nearly all 411 experiments. This step was useful in avoiding potential false positives and guided the analysis process. This first approach highlighted indirect interactions of the INSR that may be significant in podocyte biology such as CNN1, PTPN14, and MARK2 and MARK3 ( Figure 3A).

The second clustering approach focused on identifying proteins with similar temporal profiles of recruitment to the INSR upon insulin stimulation. Hierarchical clustering of the dataset was based on the normalised ion intensities (Pearson uncentred, complete linkage), and the selection of clusters of interest by finding known INSR binders in the dataset and then extracting these clusters with a correlation value no less than (0.7) ( Figure 3B). Each of the extracted clusters was interrogated against the STRING database to identify any established interactions between the cluster components, and the associated Gene Ontology terms associated with them. This approach did not yield proteins that behaved in a similar manner to some of the known INSR interactors.

**Figure 4.**
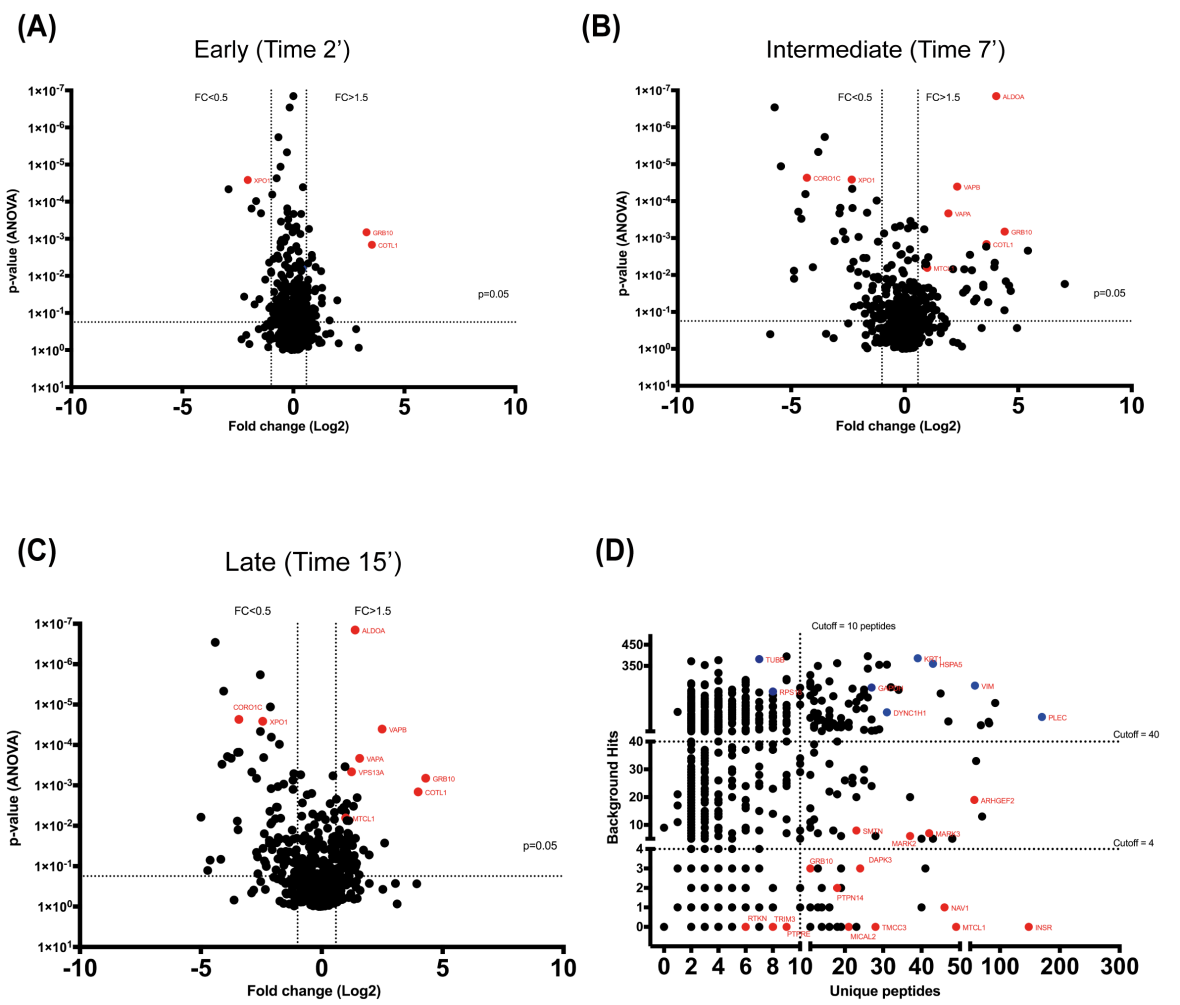
Distribution and abundance of INSR interactors. ( **A**– **C**) Volcano plots demonstrating the association/dissociation of the different proteins with the INSR at the different time points. The differences in fold change of proteins at each time point are relative to the basal condition (time =0, no insulin stimulation). ( **D**) a scatter plot detailing the chances of the detected proteins being non-specific interactors based on 411 experiment from the Contaminant Repository for Affinity Purification database, where proteins with low contaminant scores and high peptide counts are less likely to be false positive interactors.

The third approach created INSR interactor groups by applying filters. Proteins were filtered based on the number of peptides per protein (minimum of 2 or 3 unique peptides), fold change, p-value, and overlap with contaminant databases. Volcano plots of the abundance (i.e. association/dissociation with the INSR) of the different proteins across the different time points versus the significance (p-value) highlighted proteins that follow specific regulation patterns in terms of interaction with the INSR ( Figure 4A–C); for instance, VAPA and VAPB (Vesicle-associated membrane protein-associated protein A and B respectively) showed an increased association with the INSR as a function of time after insulin stimulation, and the profiles of proteins such as XPO1 (Exportin-1) and CORO1C (Coronin-1C) indicated dissociation from the INSR soon after insulin stimulation. Moreover, the Contaminant Repository for Affinity Purification dataset was utilised as a filter to reduce the chances of pursuing false positives ( Figure 4D). This analytical approach yielded a number of candidates that display specific abundance changes at the different time points including GRB10 and XPO1 at 2 minutes, VAPA and VAPB at 7 minutes and VPS13A and MTCL1 at 15 minutes.

Finally, analysis of the data using Ingenuity Pathway Analysis (IPA) was used to gain further insight into the possible connections and significant terms associated with proteins in the different sub-lists. The search resulted in highlighting multiple terms and signalling pathways that are relevant to podocyte biology and insulin signalling including the ERK/MAPK network and PI3K/AKT network (Extended data Figure 4 and 5 ^33^). The diseases and biological function top terms were mostly linked to microtubules and the actin cytoskeleton.

In summary, the combined analysis of the time course data provided insights into the temporal regulation of the INSR and the dynamic nature of protein interactions. Moreover, the varied analytical approaches were complementary and enabled us to view the data from different perspectives. The analysis process resulted in shortlisting of proteins that fall under three main categories: microtubules and actin regulation, vesicle trafficking, and kinase/phosphatase activity. The doublecortin containing protein 2 (DCDC2) was selected as a potential novel INSR interactor within the microtubule and actin regulation category, due to high abundance, response to insulin stimulation, and not reported in the Contaminant Repository for Affinity Purification of potential contaminants ^37^.

### DCDC2 co-immunoprecipitated and co-localised with the INSR

**Figure 5.**
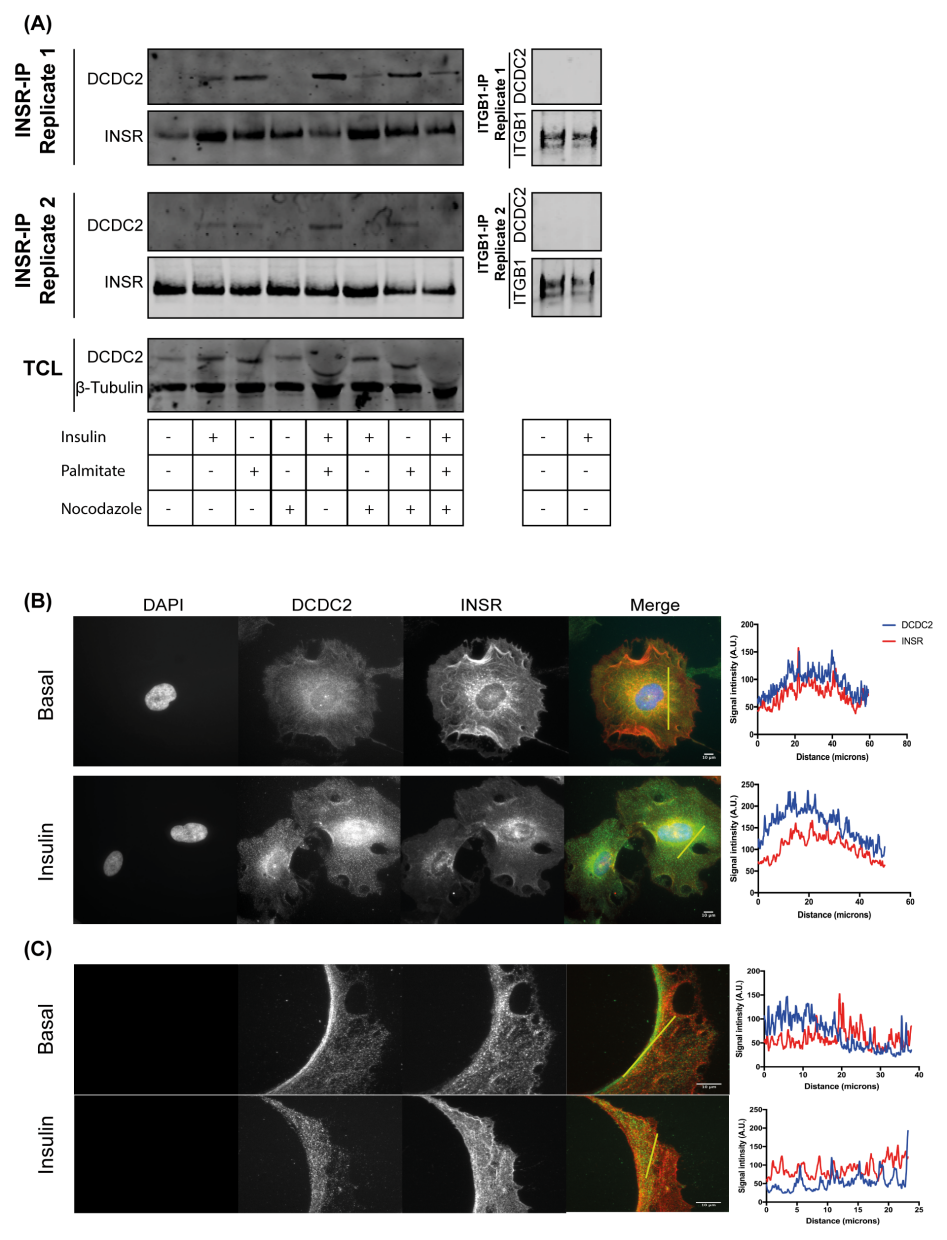
DCDC2 interacts and co-localises with the INSR in podocytes. ( **A**) DCDC2 co-immunoprecipitated with the INSR isolated from hWT-IR podocytes. Palmitate stabilised the interaction of DCDC2 with the INSR, while disrupting microtubules with nocodazole abrogated the interaction between the INSR and DCDC2. ( **B**– **C**) Immunofluorescence imaging of DCDC2 and the INSR in differentiated hWT-IR podocytes showed perinuclear co-localisation and also at the processes of the podocytes as demonstrated by the line profile. DCDC2: Doublecortin containing protein 2, ITGB1: Integrin beta 1.

To confirm the interaction of DCDC2 with the INSR, co-immunoprecipitation of DCDC2 with the INSR was performed in human wild type podocytes. DCDC2 co-immunoprecipitated with the INSR, but not integrin β1 upon insulin stimulation. Integrin β1 is not known to interact with DCDC2 and its interactome is well studied ^38^ ( Figure 5A). DCDC2 is involved with the formation of microtubules and increased expression has been associated with stabilisation of podocyte microtubules ^39^. To understand the connection between insulin signalling, and podocyte cytoskeletal remodelling in response to insulin stimulation, we used nocodazole to disrupt microtubules. Furthermore, we examined the effect of palmitate induced insulin resistance in podocytes, to examine effects on the interaction between INSR and DCDC2. The presence of palmitate enhanced the interaction between the INSR and DCDC2, while the disruption of microtubules using nocodazole abrogated this interaction. Interestingly, palmitate stabilised the interaction between of DCDC2 and INSR even after the disruption of microtubules ( Figure 5A). Immunofluorescence analysis of intensity profiles of INSR and DCDC2 showed similar distributions of elevated signal indicating close proximity of these proteins within the podocyte ( Figure 5B). In addition, focusing on cellular projections showed that the INSR and DCDC2 clustered along the projection edge, and this clustering is reduced after insulin stimulation ( Figure 5C). Overall these findings support an interaction between the INSR and DCDC2 and altered association following insulin, palmitate or nocodazole stimulation.

### DCDC2 localised to mitotic spindles and primary cilia in proliferating podocytes

To gain further insight into the localisation of DCDC2 in podocytes, we performed immunofluorescence imaging of DCDC2 in proliferating and differentiated podocytes since these cells adopt different morphologies between the differentiation states ^13^. Under proliferating conditions, the distribution of DCDC2 was uniform across the cell body with intense signals at structures resembling primary cilia and mitotic spindles (Extended data Figure 6 ^33^). This pattern became less obvious during differentiation of podocytes, where the long primary cilia structure became much smaller, and since the cells halt mitotic activity after differentiation, there were few mitotic spindles. In addition, we found that insulin stimulation initiated cortical microtubule formation, and the localisation of DCDC2 followed these microtubular changes; however, there was no significant reorganisation of DCDC2 following insulin stimulation (Extended data Figure 7 ^33^). These observations further highlight a possible role for DCDC2 in podocyte cytoskeletal function.

### DCDC2 knockdown caused distinct morphological change in podocytes

**Figure 6.**
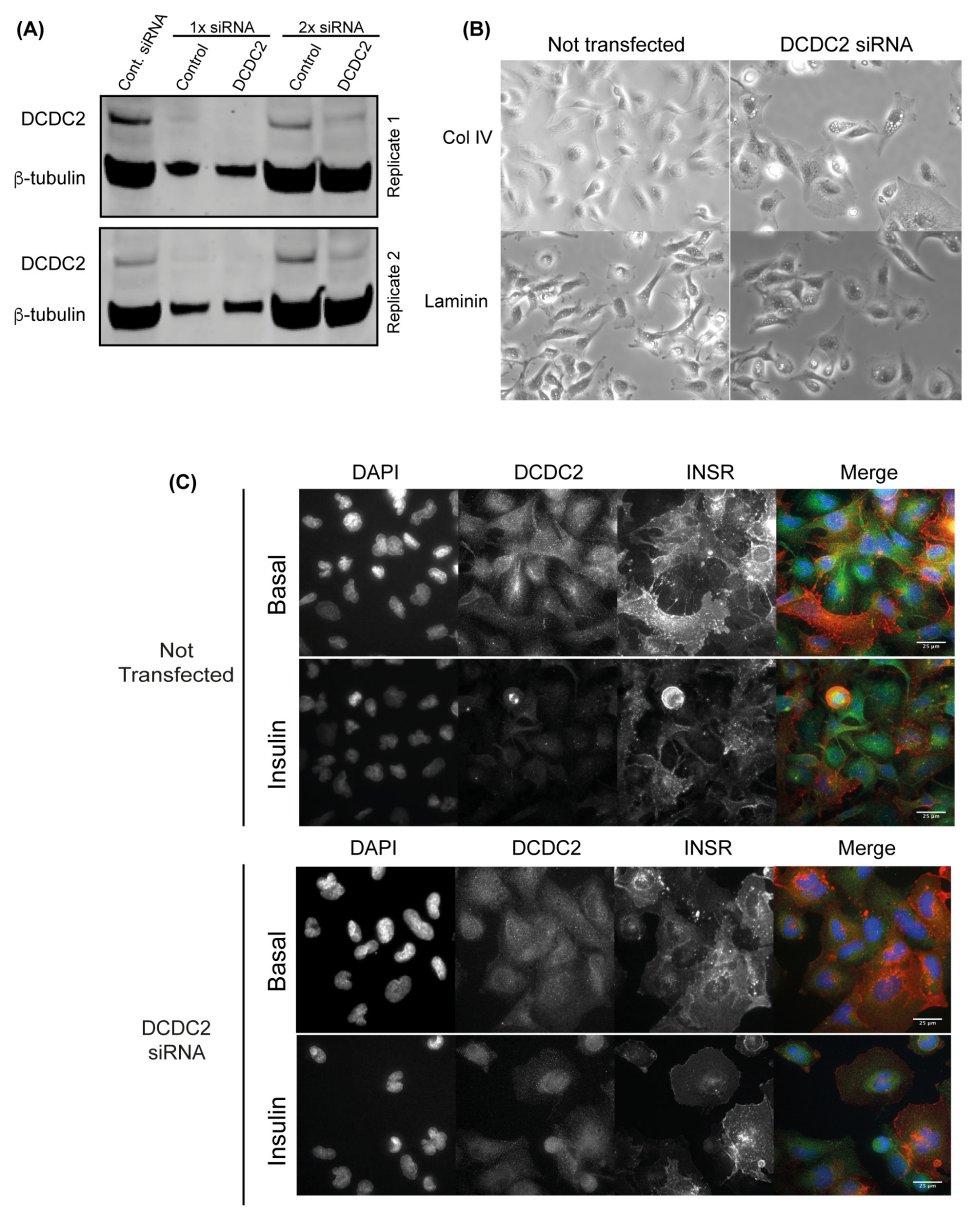
DCDC2 knockdown disrupts INSR distribution and podocyte morphology. ( **A**) DCDC2 was knocked down (kd) using pooled siRNA that achieved more than 70% reduction in the protein expression in proliferating podocytes after 2 rounds of siRNA transfection (2x siRNA). ( **B**) The morphology of DCDC2-kd podocytes differed significantly compared to normal conditions when spread on collagen IV as demonstrated by the phase contrast imaging. ( **C**) The INSR in the DCDC2-kd podocytes was localised to the plasma membrane especially after insulin stimulation. DCDC2: Doublecortin containing protein 2.

Transient knockdown of DCDC2 by siRNA in podocytes resulted in significant morphological change compared to control conditions, where knockdown cells resembled a flattened phenotype on type IV collagen and to a lesser extent on laminin ( Figure 6A and 6B). To further understand this observation in relation to the INSR, we examined the transfected cells using immunofluorescence imaging. We observed that the INSR signal was intense at the cell membrane in knockdown cells compared to control cells, which had a more uniform localisation pattern. As expected the DCDC2 signal was reduced in knockdown cells ( Figure 6C). Overall these findings reinforce a link between the INSR-DCDC2 interaction and podocyte cytoskeletal dynamics.

## Discussion

In this study we defined an *in silico* interaction network for the INSR across multiple tissues and focussed on the glomerular podocyte to identify novel INSR interactors. We confirmed that podocytes are insulin sensitive cells and become insulin resistant on stimulation with palmitate. Using INSR immunoprecipitation and MS-based proteomics we discovered DCDC2 as a novel INSR interactor in podocytes and we propose that this microtubule-associated protein may have a role in regulating the podocyte in response to insulin stimulation.

Insulin signalling is key for normal podocyte function ^11^ and it is disrupted in DKD ^2^. During the evolution of DKD, podocytes become flattened or effaced, however the underling mechanisms for this change in morphology are not well understood. This study focused on the insulin signalling pathway in podocytes at the receptor level, using affinity-enrichment over a 2–15 minute time course following insulin stimulation. The bioinformatic analysis led to the identification of doublecortin containing protein 2 (DCDC2) as a novel INSR binding protein. The rational for choosing this candidate for follow-up studies was based on the morphological change that podocytes undergo in DKD. These changes, which include foot process effacement, are associated with insulin signalling as demonstrated by podocyte-specific INSR knockout or the INSR in transgenic mice ^11^. These mice developed a phenotype that mimicked the pathological change seen in DKD, with albuminuria and podocyte effacement but in the context of normoglycemia. This observation implies that signalling from the INSR itself might be required for maintaining the cytoskeletal architecture of the podocyte, and hence DCDC2 could represent a link between the INSR and cytoskeletal regulation.

The interaction of DCDC2 with the INSR was confirmed by immunoprecipitation, and co-localisation of the two proteins was demonstrated by immunoprecipitation and imaging. Moreover, under insulin resistance conditions, DCDC2 displayed increased association with the INSR, and the disruption of microtubules led to complete loss of this interaction. DCDC2 has been linked previously to microtubule stability in podocytes ^39^; however, this is the first time that this protein has been linked to insulin signalling and the INSR. The siRNA knockdown of DCDC2 in podocytes resulted in abnormal cellular localisation of the INSR, compared to controls, together with morphological changes. These observations suggest that the relation between DCDC2 and INSR could be related to intracellular trafficking especially following insulin stimulation. The hWT-IR cell line did not become insulin resistant upon stimulation with palmitate and this may be related to high levels of the INSR due to overexpression. However, the presence of palmitate also had a clear effect on the interaction between DCDC2 and INSR. One explanation for this observation is that the DCDC2 interaction with the INSR is stabilised by palmitate; tipping the balance between the association and dissociation state.

DCDC2 in the context of kidney diseases is linked to renal ciliopathies and nephronophthisis; however, insulin signalling is not directly linked to these disorders ^40, 41^. The detection of DCDC2 at structures that resembled primary cilia and also at mitotic spindles by immunofluorescence imaging indicates that DCDC2 might play multiple biological functions in proliferating podocytes. These structures are not observed in differentiated podocytes, and DCDC2 does not seems to re-localise in response to insulin signalling. Podocytes have been reported to adopt different morphologies when seeded onto different extracellular matrix ligands ^42^; however, the knockdown of DCDC2 resulted in significant morphological changes when the cells were plated on laminin or type IV collagen compared to the controls. This indicates the possibility of a role of DCDC2 in specific protein networks that dictate the podocyte morphological adaptation in response to outside-in signalling and might represent a convergence with insulin signalling.

In conclusion, this study provides insight into both the complexity and specificity of insulin signalling in podocytes. Furthermore, it may explain how insulin resistance can affect the integrity of the glomerular filtration barrier in kidney disease associated with and insulin resistant states, such as DKD. The bioinformatic analysis of the INSR complex led to the discovery of expected and novel INSR interactors and confirmation of DCDC2 as a novel INSR binding protein. Further investigation of the link between the insulin signalling, DCDC2 and podocyte cytoskeletal regulation may lead to improved understanding about mechanisms that underlie podocyte dysfunction in DKD.

## Data availability

### Underlying data

Figshare: Proteomic analysis of insulin receptor interactors in glomerular podocytes. https://doi.org/10.6084/m9.figshare.c.5015879.v4 ^33^

This project contains the following underlying data:

- Final Supp data Progenesis QI INSR_TIME_COURSE.xlsx (Progenesis QI output files)
  - Progenesis QI INSR_TIME_COURSE.xlsx (Progenesis QI output files)
  - Scaffold proteomics INSR_TIME_COURSE.xls (Mascot and Scaffold analysis output as spectral counts)
  - 20161125_RL_SH_02.raw (INSR IP LC-MSMS data RAW files time 0 min)
  - 20161125_RL_SH_03.raw (INSR IP LC-MSMS data RAW files time 0 min)
  - 20161125_RL_SH_04.raw (INSR IP LC-MSMS data RAW files time 0 min)
  - 20161125_RL_SH_05.raw (INSR IP LC-MSMS data RAW files time 0 min)
  - 20161125_RL_SH_06.raw (INSR IP LC-MSMS data RAW files time 2 min)
  - 20161125_RL_SH_07.raw (INSR IP LC-MSMS data RAW files time 2 min)
  - 20161125_RL_SH_08.raw (INSR IP LC-MSMS data RAW files time 2 min)
  - 20161125_RL_SH_09.raw (INSR IP LC-MSMS data RAW files time 2 min)
  - 20161125_RL_SH_10.raw (INSR IP LC-MSMS data RAW files time 7 min)
  - 20161125_RL_SH_11.raw (INSR IP LC-MSMS data RAW files time 7 min)
  - 20161125_RL_SH_12.raw (INSR IP LC-MSMS data RAW files time 7 min)
  - 20161125_RL_SH_11_161126202211_15.raw (INSR IP LC-MSMS data RAW files time 7 min)
  - 20161125_RL_SH_12_161126222443_16.raw (INSR IP LC-MSMS data RAW files time 15 min)
  - 20161125_RL_SH_13_161127002717_17.raw (INSR IP LC-MSMS data RAW files time 15 min)
  - Figure 2 (Original western blotting)
  - Figure 2 (Original IP)
  - Figure 5 (Original IF Images)
  - Figure 5 (Original western blotting and IP)
  - Figure 6 (Original IF Images)
  - Figure 6 (Original western blotting)
  - Figure 6 (Phase contrast images)
  - Extended data - Figure 2 (Original western blotting)
  - Extended data - Figure 3 (Original western blotting and IP)
  - Extended data - Figure 6 (Original IF Images)
  - Extended data - Figure 7 (Original IF Images)

Figshare: Proteomic analysis of insulin receptor interactors in glomerular podocytes. https://doi.org/10.6084/m9.figshare.c.5015879.v4 ^33^

This project contains the following extended data:

- Extended data figures.pdf (Extended data Figures)
- ExtendedDataTable1.pdf (Extended data – Table 1)
- ExtendedDataTable2.pdf (Extended data – Table 2)

**Extended data Table 1.** Time course insulin stimulation. Podocytes were stimulated with 100 nm insulin for 2,7 and 15 minutes. Total spectral counts (TSC) are indicated for each replicate sample. Molecular Weight = MWt, kilo Dalton = kDa, Minutes = '.

**Extended data Table 2.** The insulin receptor and 26 of its known interactors that were also found in the INSR in silico network. These 26 interactors were found across two independent experiments. 

**Extended data Figure 1.** (A) Literature known insulin receptor interactors form a highly interconnected network. (B) Gene Ontology (GO) analysis of the components of this network highlighted expected terms such as protein tyrosine kinase activity, receptor tyrosine kinase binding, and protein tyrosine phosphatase activity.

**Extended data Figure 2.** (A) Human wild type podocytes over expressing the insulin receptor (hWT-IR) are insulin responsive and yielded good amounts of the INSR with immunoprecipitation. (B) hWT-IR cells responded to insulin stimulation with an increase in glucose uptake but they did not become insulin resistant in the presence of palmitate. (C) Co-IP of the INSR and some of its known interactors was reproducible in the hWT-IR cells.

**Extended data Figure 3**. (A) an illustration of the workflow for the generation of the main proteomic dataset. (B) Immunoprecipitation of the INSR from hWT-IR podocytes at different time points demonstrating co-immunoprecipitation of phospho-IRS1. (C) The mapping of detected known INSR interactors from two experiments onto the *in silico* INSR network.

**Extended data Figure 4.** Ingenuity pathway analysis (IPA) was used to gain further insight into the possible connections and significant terms associated with proteins in the different sub-lists. (A) High level view of signalling pathways altered across the early (2 minute), intermediate (7 minute) and late (15 minute) time points. (B) Genes is the signalling by Rho family GTPases pathway. (C) Genes in the RhoA signalling network. (D) Genes in the ERK/MAPK signalling network and (E) Genes in the PI3K/AKT signalling network.

**Extended data Figure 5.** Ingenuity pathway analysis (IPA) was used to gain further insight into the possible connections and significant terms associated with proteins in the different sub-lists. (A) High level view of diseases and biological function pathways altered across the early (2 minute), intermediate (7 minute) and late (15 minute) time points. (B) Genes in the quantity of actin stress fibres cluster. (C) Genes in the quantity of actin filaments cluster.

**Extended data Figure ‎6**: DCDC2 co-localised with microtubules and clustered in primary cilia and mitotic spindles. (A) The immunofluorescence of DCDC2 demonstrated the cellular distribution and co-localisation with microtubules, primary cilia, and mitotic spindles in proliferating hWT-IR podocytes. (B) DCDC2 shows a slight overlap with the insulin receptor that does not change with insulin stimulation. (40x magnification).

**Extended data Figure 7:** DCDC2 co-localised with microtubules in differentiated human podocytes. (A) The immunofluorescence of DCDC2 demonstrated a cellular distribution resembling microtubular filaments. (B) DCDC2 and microtubules co-staining demonstrated the co-localisation of the two proteins, and insulin stimulation did not influence the localisation DCDC2. 

Data are available under the terms of the Creative Commons Zero "No rights reserved" data waiver (CC0 1.0 Public domain dedication).
