# Supplementary material for: Global proteomic analysis of insulin receptor interactors in glomerular podocytes
Source: Wellcome Open Res. 2020 Aug 26;5:202. [Version 1] doi: 10.12688/wellcomeopenres.16072.1 (PMC7788524; doi:10.12688/wellcomeopenres.16072.1)
Supplement: Peer review history

# Peer review: Reviewer response for version 1

**DOI:** 10.21956/wellcomeopenres.17634.r41679

**Published:** 2021-01-06

**Authors:** Marco Prunotto

**Affiliations:** 1 School of Pharmaceutical Sciences, Institute of Pharmaceutical Sciences of Western Switzerland, University of Geneva, Geneva, Switzerland

A very solid paper by the well known group of Rachel Lennon in Manchester. The works builds on prior proteomics works published in top nephrology journals

 This study provides insight into the complexity of insulin receptor interactors in key glomerular cells at the core of the filtration activity of the kidney, the podocytes pointing out, amongst all, to the DCDC2 protein. This is an interesting and novel finding that might unveil a novel intervention axis to modulate podocyte biology.

Is the work clearly and accurately presented and does it cite the current literature?

Yes

If applicable, is the statistical analysis and its interpretation appropriate?

Yes

Are all the source data underlying the results available to ensure full reproducibility?

Yes

Is the study design appropriate and is the work technically sound?

Yes

Are the conclusions drawn adequately supported by the results?

Yes

Are sufficient details of methods and analysis provided to allow replication by others?

Yes

Reviewer Expertise:

Drug discovery and development.

I confirm that I have read this submission and believe that I have an appropriate level of expertise to confirm that it is of an acceptable scientific standard.

# Peer review: Reviewer response for version 1

**DOI:** 10.21956/wellcomeopenres.17634.r40168

**Published:** 2020-09-21

**Authors:** Paul Brinkkoetter

**Affiliations:** 1 Faculty of Medicine, University Hospital Cologne, Cologne, Germany

The manuscript entitled "Global proteomic analysis of insulin receptor interactors in glomerular podocytes“ by Hosawi and colleagues investigates the interacting proteins of the insulin receptor in glomerular podocytes. Podocytes which make up the third outer layer of the glomerular filtration barrier are of critical importance to maintain the renal filter apparatus. Previous studies have identified the cells to be direct targets of insulin. In diabetic kidney disease insulin signaling events contribute to the observed progressive podocyte dysfunction. In this study, the authors employed a well-characterized human podocyte cell line and isolated the insulin receptor and its interacting proteins by immunoprecipitation followed by label-free mass spectrometry. Doing so, the authors identified known and novel interacting proteins of the INSR. Additional *in vitro* studies on one of the novel interacting proteins, i.e. DCDC2, further emphasized the importance of this metabolic signaling cascade.

 This is an elegant and well-performed study out of one of the leading labs in the field on metabolic signaling at the glomerular filtration barrier. The manuscript characterized the INSR interactome in glomerular podocytes and identified novel, interesting candidates to be studied further. I have only minor concerns/comments. The study is primarily based on human immortalized human podocytes with and without overexpression of the human insulin receptor. Additional co-IP studies using freshly isolated glomerular lysates would greatly strengthen the main findings.

Is the work clearly and accurately presented and does it cite the current literature?

Yes

If applicable, is the statistical analysis and its interpretation appropriate?

Yes

Are all the source data underlying the results available to ensure full reproducibility?

Yes

Is the study design appropriate and is the work technically sound?

Yes

Are the conclusions drawn adequately supported by the results?

Yes

Are sufficient details of methods and analysis provided to allow replication by others?

Yes

Reviewer Expertise:

Glomerular biology.

I confirm that I have read this submission and believe that I have an appropriate level of expertise to confirm that it is of an acceptable scientific standard.
